# Supplementary material for: Parents of children with hearing loss: Impact and exposure of COVID-19 on mental health
Source: PLoS One. 2023 May 2;18(5):e0285249. doi: 10.1371/journal.pone.0285249 (PMC10153707; doi:10.1371/journal.pone.0285249)
Supplement: Attachment — Submitted filename: PLOS One Response to Reviewers.docx [file pone.0285249.s002.docx]

| **Journal Requirements** | | |
| --- | --- | --- |
| Please ensure that your manuscript meets PLOS ONE's style requirements, including those for file naming | We have reviewed the requirements for PLOS One and followed accordingly. | N/A |
| Ethics statement: Did participants provide their written or verbal informed consent to participate in this study? | The first page of the questionnaire contained the informed consent form. Clicking “yes” indicated participant consent to participate. | 119-123 |
| Ethic statement: If consent was verbal, please explain i) why written consent was not obtained, ii) how you documented participant consent, and iii) whether the ethics committees/IRB approved this consent procedure. | Consent was documented within the electronic questionnaire. If participant clicked “no” to agreeing to participate, the questionnaire immediately ended. The University of Miami IRB approved this consent procedure. | 119-123 |
| We note that the grant information you provided in the ‘Funding Information’ and ‘Financial Disclosure’ sections do not match. When you resubmit, please ensure that you provide the correct grant numbers for the awards you received for your study in the ‘Funding Information’ section | We have reviewed funding information and ensured that reporting of funding is consistent. | N/A |
| We note that you have indicated that data from this study are available upon request. PLOS only allows data to be available upon request if there are legal or ethical restrictions on sharing data publicly. For more information on unacceptable data access restrictions, please see http://journals.plos.org/plosone/s/data-availability#loc-unacceptable-data-access-restrictions. | De-identified dataset has been uploaded as supporting information. | N/A |
| Revised Cover Letter: Please address If there are ethical or legal restrictions on sharing a de-identified data set, please explain them in detail (e.g., data contain potentially sensitive information, data are owned by a third-party organization, etc.) and who has imposed them (e.g., an ethics committee). Please also provide contact information for a data access committee, ethics committee, or other institutional body to which data requests may be sent. | Cover letter has been revised. We have also uploaded a de-identified dataset according to the journal policies. | Cover letter |
| Revised Cover Letter: Please address If there are no restrictions, please upload the minimal anonymized data set necessary to replicate your study findings as either Supporting Information files or to a stable, public repository and provide us with the relevant URLs, DOIs, or accession numbers. For a list of acceptable repositories, please see http://journals.plos.org/plosone/s/data-availability#loc-recommended-repositories. | A de-identified dataset has been uploaded as supporting information and this has also been clarified in the revised cover letter. | Cover letter |
| Please include your full ethics statement in the ‘Methods’ section of your manuscript file. In your statement, please include the full name of the IRB or ethics committee who approved or waived your study, as well as whether or not you obtained informed written or verbal consent. If consent was waived for your study, please include this information in your statement as well. | We have revised the Method section to provide more detail regarding our consent process. Consent was documented within the electronic questionnaire. If participant clicked “no” on the consent page the questionnaire immediately ended. The University of Miami IRB approved this consent procedure. | 119-123 |
| **Reviewer 1** | | |
| In terms of content, it would be interesting if some aspects were more investigated. For example, the introduction is a bit repetitive and not very in-depth. | Thank you for the suggestion. We rewrote the introduction for better clarity and flow. | Introduction |
| As far as the survey is concerned, some items could be reported as examples (perhaps the most significant ones or those with the most relevant scores). | All items are listed in Figure 1, however, as requested we included a few examples within the text. | 147-160 |
| Within the limits of the work, it is pointed out that it was not possible to retrieve data on children's audiometric thresholds. In addition to these data, however, it would have been important to have information regarding the etiology of hearing loss, any associated disabilities, the progress of rehabilitation (perceptual and language outcomes), perhaps even to know how long the child has been wearing the device, whether it is a prosthetic or cochlear implant. The "child device type" alone is likely to be a variable that does not allow for in-depth analysis. | We thank the reviewers for their thoughtful reviews. We agree having more in-depth audiological information would have been helpful. In Table 1 the percent of children who wore cochlear implants versus hearing aids or bone conduction devices was reported. Given our limitations with the electronic survey and parent report, we were unable to obtain more in-depth audiological information as parents often are unable to report this information independently of their providers. We have added this to our limitations section and also as a future direction. | 397-401 |
| Equally interesting would be to have the same data for a sample of parents of children without hearing impairment in order to see how much the children's condition affects the parent's status at such a critical time | We agree this would have been an interesting comparison, however, recruitment from our study was via our clinical database as part of our pediatric hearing loss program which we have described in the Method section. | 117-118 |
| When in the discussion of making a comparison with similar assessments of parents of normal hearing children (e.g., "This is more than three times higher than the estimated prevalence of 18% in the general population"), what tools were used in the cited work? Does this refer to a clinical diagnosis of depression? | We have clarified when references for prevalence rates were based on clinical diagnoses. In addition, we also clarified that the references study comparing pre to post COVID rates of depression also used the same questionnaire used in the current study, the Patient Health Questionnaire. | 261-272 |
| Lines 221-224 should be more clearly analyzed on the one hand the relationship between general population and parents involved in the study and, on the other hand, between the pre- and post-covid situation, and then discuss the data together. | We have clarified our discussion of studies comparing the general population versus studies reporting on changes in depression prior and during the pandemic. | 327-338 |
| Lines 282-303 better argue the discussion; at some points, it does not appear to be sufficiently supported by the results. | We have reworded some of our discussion to ensure that our conclusions are more supported by our results and specific to our sample of parents of children with hearing loss. | 340-364 |
| Why did only one parent per child participate in the study? It would also be interesting to see if there are differences between the different parental figures. | Differences in parental perspective (as it pertains to depression, anxiety, and post-traumatic stress) were unable to be evaluated. Our patient listserv only includes the contact information for the primary caregiver. We have specified this more clearly in the Method section. | 117-118 |
| It would be better to define the acronym Post-traumatic stress disorder after the first time the acronym is reported. | Thank you for your careful review, we have made this change. | 77 |
| **Reviewer 2** | | |
| A major concern with this paper is that the aims and how the IV and DVs are measured is not always clear. At various points the authors refer to the construct of "well being" but this is not clearly defined, so it is unclear whether this is referring to "mental health" or to the construct of well being as measured by items from the CEFIS. Your aims are to measure “impact and exposure” of COVID 19 (CEFIS scales) on parent mental health (GAD7, PHQ, IESR) so it is very confusing to conflate mental health measures with “well being” when CEFIS items directly assess questions related to well being (which is measured by their part 2- impact scale). | We appreciate Reviewer’s 2 critical review. We have changed the wording of “well-being” to “mental health” throughout the manuscript to provide clarity and consistency to the aims, IVs and DVs. | Throughout manuscript |
| Similarly, in the literature review, papers referring to parental depression are instead described as assessing "well being." This needs to be clarified and corrected throughout the paper. | We agree and have made changes throughout the manuscript to clarify the aims, IVs and DVs. | Throughout manuscript |
| A striking example of this confusion of terms comes from Lines 74-77:  " This study evaluates the impact (WELLBEING, emotional adjustment, and family relationships) and exposure 75 (school closures, changes in housing, changes in employment) of COVID-19 on the overall 76 WELLBEING of parents of children with hearing loss, as defined by symptoms of depression, 77 anxiety, and post-traumatic stress disorder.  This would suggest that you are measuring wellbeing as both an IV and DV? | As requested we have made changes to the wording throughout the manuscript. | Throughout manuscript |
| Another limitation of this study is that the CEFIS impact scales have items measuring anxiety. Therefore, findings regarding linear regression that were found to be significant between the GAD7 and CEFIS impact scales may reflect overlapping constructs rather than overall "impact" predicting anxiety per se. | The reviewer has a valid point; however, it should be noted that while there is a question on anxiety in the CEFIS impact scale, this scale is intended to measure overall perceived impact of COVID-19, while the GAD-7 is intended to measure generalized anxiety symptoms. Although we expect there to be some overlap, they are assessing different constructs. These measures have also been used together in previous studies (Graziano et al., 2022; Noiji et al., 2022). | N/A |
| Furthermore, it really needs to be clarified whether you are using the CEFIS to measure 3 independent variables (exposure and impact) or 3 (exposure, impact, and distress). If I’m reading this correctly, after looking closely at the CEFIS measure, it seems like you are measuring parent distress and child distress based on a SINGLE item from the CEFIS? This does not seem like one of your initial aims and seems methodologically flawed. From line 34: "In addition, both impact and exposure predicted COVID related parental distress, while child distress was only affected by impact of COVID-19"  The following lines seem to conflate impact and distress in a way that is confusing:  Lines 118 -120…." open-ended question inquiring about the family’s experience and effects of COVID. Higher scores on both the exposure and impact scales denote more negative exposure and impact/distress. (Are these the same construct? or different constructs?). | Distress is listed in our aims, “Lastly, we predicted that impact of COVID-19 would be associated with parental distress.” Additionally, the single question of parent distress has been shown to be a valid measure of distress (Enlow et al., 2022; https://www.ncbi.nlm.nih.gov/pmc/articles/PMC8755387/). We have clarified this in the methods section. Methods have been revised for clarity. Only the exposure scale, impact scale, and distress questions were used in this study. We did not use the open-ended question inquiring about family experience. | 154-155; 158-160 |
| In general, I find the interpretation of data related to child distress to be weak given the limited data available to adequately assess child distress and since this is not a primary aim of the study, I would recommend dropping this discussion from the paper | Based on the reviewer comments, we have removed the results related to child distress as it was not our primary aim. | Throughout manuscript |
| A methodological limitation of the study not well discussed in the discussion is the availability of the survey for a very short window (one month) at the beginning of COVID19 (June 9th, 2020- July 9th 91 , 2020) at the height of uncertainty related to COVID. There is little discussion about why this window was chosen. | We have addressed this in our limitations. The beginning of the pandemic was an uncertain time and the authors did not know how long the pandemic would last. Therefore, a short window with enough time to gather an ample sample size was chosen. If the length would have been extended for months, there may have been significant differences between parent perceptions at the beginning of the survey window versus the end. In hindsight, this allowed for a more consistent evaluation. | 387-394 |
| It would have been of more practical value to reassess respondents at a later timepoint in order to better ascertain the persistence of these elevated symptoms. Failure to do this means caution must be used in drawing conclusions from this data particularly when extrapolating beyond the data regarding suggested implications for intervention (e.g., lines 309-310 regarding necessity for telehealth). Discussion sections such as this should be edited to more clearly delineate what can be clearly drawn from the actual data presented, versus possible implications. | We have softened the language to not overly draw conclusions that are not supported by our results. Our data does show elevations in depression and anxiety, thus, supporting the need for more mental health services for parents of children with hearing loss. In our clinic and with the hearing loss population, telemedicine has been a valuable addition to our service delivery allowing easy access to our psychology and social work team. | Discussion |
| Some of the interpretation of the data is somewhat misleading. For example, the authors cite that " 20% of the participants endorsed PTSD symptoms above the clinical concern 168 range on the IES, with 10% scoring in the highest range indicating that symptoms are high 169 enough to suppress immune functioning and warrant further evaluation and intervention." This is not adequately captured in the TABLE 2 where the mean score is reported as falling below the clinically significant range for PTSD (WHICH IS NOT DISCUSSED) but there is quite a range | We have clarified within the text that the means for the sample were within the normal range. However, it is still important to highlight the percentages of individuals scoring within elevated ranges. This is common practice in the psychological and clinical literature. Table 2 reports both the mean and the minimum and maximum scores for each measure. The table note also specifies the scores and their descriptive clinical classifications. | 203-210 |
| This seems to misrepresent or not adequately capture the data in its entirety. What you’re finding here is that the average is below the clinical cut off, and with this small sample size that 20% falling above the clinically significant range is harder to interpret. It’s also unclear to me whether the Min-Max represents min-max possible scores or the range of scores for this sample- please consider relabeling. | Hearing loss is a low incidence population and our sample is representative of studies reporting on this special population. We have expanded our results to report the mean scores, while also highlighting the percentage of parents scoring within the elevated ranges. The Min-Max scores reported in Table 2 are for the sample. | 203-210; Table 2 |
| The literature review and discussion sections should be edited for clarity. The authors attempt to weave together literature that children who are DHH are more at risk for mental health concerns, that parents of DHH children are more vulnerable to parenting stress and depression impacting caregiver sensitivity and language outcomes, and that children of depressed parents are more vulnerable to poor treatment adherence. However, both the literature review and discussion sections, appear disjointed. | We greatly appreciate your suggestion and both sections have been re-worded for clarity and improved flow. | Introduction and Discussion |
| This is especially true in the discussion section where the authors link findings that in the general population parental depression is correlated with missed appointments and findings that poor hearing device compliance is linked with poor outcomes. (231-236) However, a transitive property is being utilized here that was not directly measured in this study. (If data regarding treatment adherence during this time period was available, it would strengthen the paper). As presented, this feels like an overextension and should be presented as a possible concern to be explored in future research (e.g., it's unclear whether elevated depressive symptoms during a pandemic, especially when it is unclear how persistent those symptoms are, will have same implications for treatment adherence as past research on parents with depression meeting clinical criteria for depression). | We have softened our conclusions and also added as a limitation and future direction | 397-401 |
| Line 71-73"As the world prepares for post-pandemic, it is crucial that we begin to identify and understand how mental health has been affected families of children with hearing loss in order to influence our clinical recommendations and guide our medical treatment decisions.” | We have revised the sentence for grammatical accuracy. | 368-375 |
| Line 246 -249 ..."system. Further, PTSD can get worse if not addressed, resulting in substantial distress and disruption of social and occupational functioning. According to the CDC those with multiple stressors or persons with chronic medical illnesses are at greater risk for developing long-term reactions, such as PTSD [37]" (latter sentence here may be better placed before the first?) | As suggested, we have changed the order of the sentences to improve clarity. | 288-295 |
