# Supplementary material for: Parents of children with hearing loss: Impact and exposure of COVID-19 on mental health
Source: PLoS One. 2023 May 2;18(5):e0285249. doi: 10.1371/journal.pone.0285249 (PMC10153707; doi:10.1371/journal.pone.0285249)
Supplement: Attachment — Submitted filename: Response to ReviwersFinal.docx [file pone.0285249.s003.docx]

| **Reviewer 1 Comments** | **Response** | **Location of Change** |
| --- | --- | --- |
| In any case, the authors have better pointed out these gaps and highlighted the study's weaknesses, to be resolved perhaps by further studies.  Regarding especially the absence of correlations between questionnaire scores and children's audiological variables, it is obvious how these may be impacted by the limitations imposed by the pandemic. But my comment in the previous review was not so much about the influence of the pandemic on these variables, but the opposite, namely the possible mediating role that these variables may have on parental mental health. In fact, I imagine that the child's rehabilitation (audiological outcome, level of compensation of hearing loss, time of device use, results of language rehabilitation, child's listening ability, frequency of clinical follow-ups...) are closely related to the consequences that all covid-19 limitations had on the well-being of the parents of these children, and are therefore inescapable variables in the perspective of a more correct analysis of the results.  Therefore, I would better emphasize this among the limitations and future prospects of the study. | As suggested by Reviewer 1, we have expanded our limitations and future research to include the potential mediational effects of audiological data and/or rehabilitation on parental wellbeing. | 346-350 |

| **Reviewer 2 Comments** | **Response** | **Location of Change** |
| --- | --- | --- |
| Lines 75-77 : This study evaluated the impact (MENTAL HEALTH, emotional adjustment, and family relationships) and exposure (school closures, changes in housing, changes in employment) of COVID-19 on the overall mental health of parents of children with hearing loss, as defined symptoms of depression, anxiety, and Post-Traumatic Stress Disorder (PTSD).  - These lines should be rewritten. As written, it sounds like you are looking at relationships between Impact (MENTAL HEALTH)…. “on the overall MENTAL HEALTH” of parents of children with HL. In the text beginning on line 152 impact is summarized as “individuals’ overall wellbeing, emotional adjustment, sleeping, and ability to care for their children”- please edit for consistency | We have edited the sentence for consistency between the introduction and method. | 65-66 |
| Also lines 158-161 add further confusion by adding in “distress” which is not mentioned earlier and seems to introduce a 3rd construct | Distress is consistently mentioned throughout the manuscript. In fact, on line 75 in the introduction we mention our hypothesis that , “…impact of COVID-19 would be associated with parental distress.” The distress scale that was utilized is part of the CEFIS measure, which is discussed in the measures section. | NA |
| Line 142 – spell out acronym first (not just in the header) “The CEFIS” | We have spelled out the acronym, CEFIS. | 107 |
| Line 342 “Thus, it appears that for our specialized population of parents of children with hearing, …” (insert …children with (reduced?) hearing??? Or “children with hearing (loss?)) | Thank you for your thorough review, this was a typo and we have revised the sentence. | 292 |
